# SAMHD1 Phosphorylation Coordinates the Anti-HIV-1 Response by Diverse Interferons and Tyrosine Kinase Inhibition

**DOI:** 10.1128/mBio.00819-18

**Published:** 2018-05-15

**Authors:** Matthew A. Szaniawski, Adam M. Spivak, James E. Cox, Jonathan L. Catrow, Timothy Hanley, Elizabeth S. C. P. Williams, Michel J. Tremblay, Alberto Bosque, Vicente Planelles

**Affiliations:** a Division of Microbiology and Immunology, Department of Pathology, University of Utah School of Medicine, Salt Lake City, Utah, USA; b Department of Medicine, University of Utah School of Medicine, Salt Lake City, Utah, USA; c Department of Biochemistry, University of Utah School of Medicine, Salt Lake City, Utah, USA; d Axe des Maladies Infectieuses et Immunitaires, CR-CHU de Québec-Université Laval, Quebec City, QC, Canada; e Département de Microbiologie, Infectiologie et Immunologie, Université Laval, Quebec City, QC, Canada; f Department of Microbiology, Immunology, and Tropical Medicine, George Washington University, Washington, DC, USA; University of Washington

**Keywords:** CDK1, dasatinib, interferon, human immunodeficiency virus, macrophages

## Abstract

Macrophages are susceptible to human immunodeficiency virus type 1 (HIV-1) infection despite abundant expression of antiviral proteins. Perhaps the most important antiviral protein is the restriction factor sterile alpha motif domain and histidine/aspartic acid domain-containing protein 1 (SAMHD1). We investigated the role of SAMHD1 and its phospho-dependent regulation in the context of HIV-1 infection in primary human monocyte-derived macrophages and the ability of various interferons (IFNs) and pharmacologic agents to modulate SAMHD1. Here we show that stimulation by type I, type II, and to a lesser degree, type III interferons share activation of SAMHD1 via dephosphorylation at threonine-592 as a consequence of signaling. Cyclin-dependent kinase 1 (CDK1), a known effector kinase for SAMHD1, was downregulated at the protein level by all IFN types tested. Pharmacologic inhibition or small interfering RNA (siRNA)-mediated knockdown of CDK1 phenocopied the effects of IFN on SAMHD1. A panel of FDA-approved tyrosine kinase inhibitors potently induced activation of SAMHD1 and subsequent HIV-1 inhibition. The viral restriction imposed via IFNs or dasatinib could be overcome through depletion of SAMHD1, indicating that their effects are exerted primarily through this pathway. Our results demonstrate that SAMHD1 activation, but not transcriptional upregulation or protein induction, is the predominant mechanism of HIV-1 restriction induced by type I, type II, and type III IFN signaling in macrophages. Furthermore, SAMHD1 activation presents a pharmacologically actionable target through which HIV-1 infection can be subverted.

## IMPORTANCE

Our experimental results demonstrate that SAMHD1 dephosphorylation at threonine-592 represents a central mechanism of HIV-1 restriction that is common to the three known families of IFNs. While IFN types I and II were potent inhibitors of HIV-1, type III IFN showed modest to undetectable activity. Regulation of SAMHD1 by IFNs involved changes in phosphorylation status but not in protein levels. Phosphorylation of SAMHD1 in macrophages occurred at least in part via CDK1. Tyrosine kinase inhibitors similarly induced SAMHD1 dephosphorylation, which protects macrophages from HIV-1 in a SAMHD1-dependent manner. SAMHD1 is a critical restriction factor regulating HIV-1 infection of macrophages.

## INTRODUCTION

Macrophages are major targets of human immunodeficiency virus type 1 (HIV-1), and their importance in infection establishment, progression, and persistence has been studied at length (1, 2). They are thought to contribute importantly to virus amplification and dissemination during primary infection, and in the central nervous system (CNS), HIV-1-infected macrophages and microglia precipitate a spectrum of neurological impairments that can persist in the setting of antiretroviral therapy (ART) (3–5). It is known that macrophages exhibit low permissiveness to infection, in part due to the constitutive expression of the antiviral restriction factor sterile alpha motif (SAM) domain- and histidine/aspartic acid (HD) domain-containing protein 1 (SAMHD1), a deoxynucleoside triphosphate (dNTP) triphosphohydrolase that restricts HIV-1 by maintaining dNTP concentrations below the threshold required for efficient reverse transcription (6–9).

Interferons (IFNs) play an important role in preventing viral infection through multiple effector mechanisms. Numerous studies have pointed to enhanced expression of various restriction factors that act at multiple stages of HIV-1 infection, including MX2, GBP5, APOBEC3A, and others, to explain the potent antiviral effects of IFNs (10–14). In this context, activation of the interferon regulatory factor (IRF) family of transcription factors, nuclear translocation, and recognition of a conserved DNA sequence known as an IFN-stimulated response element (ISRE) present in the promoter-enhancer regions of a number of genes direct these antiviral programs (15, 16). Though alpha interferon (IFN-α) and IFN-β are perhaps the most well-studied type I IFNs, numerous others exist in humans including IFN-ε, whose relevance in HIV-1 restriction has been demonstrated recently in macrophages (17). The sole member of the type II IFN family, IFN-γ, is widely recognized for its role in macrophage activation, and it was recently shown to induce a potent, Env-dependent block to HIV-1 infection in CD4^+^ T cells that was distinct from that induced by type I IFN (18). The type III IFNs, which include IFN-λ1, -λ2, and -λ3, have been shown to exhibit anti-HIV-1 activity, and may be relevant in specific tissue sites such as the vaginal mucosa (19). Macrophages are highly responsive to the modulating effects of many diverse interferons and interferon-like molecules; however, the mechanisms underlying the potent anti-HIV-1 capacities of diverse IFNs remain incompletely defined (20). SAMHD1 is regulated at the level of phosphorylation by type I interferon, but to what extent type II and III IFNs affect SAMHD1 phosphorylation and whether SAMHD1 is required for their activity remain unknown (21).

In this study, we examined the abilities of divergent IFN families to inhibit HIV-1 infection in primary human monocyte-derived macrophages (MDM) and provide mechanistic insight into the major effector function of type I, type II, and type III IFNs. We extended the study to explore the HIV-1 restriction potential of a panel of FDA-approved tyrosine kinase inhibitors (TKI) in an effort to define SAMHD1 as a potential pharmacologic target for anti-HIV-1 efforts. These studies show that SAMHD1 serves as the key regulator of HIV-1 infectivity in MDM whose activity is rapidly induced at the level of protein dephosphorylation.

## RESULTS

### Type I, type II, and type III IFNs induce SAMHD1-T592 dephosphorylation, resulting in enhanced restriction of HIV-1 infection in MDM.

**FIG 1:**
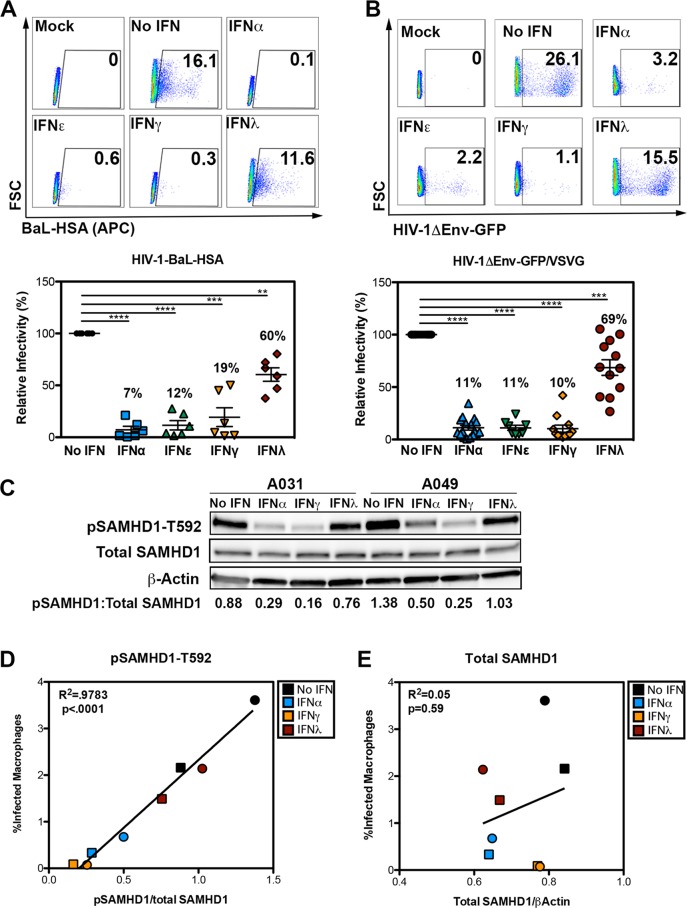
Diverse IFNs restrict HIV-1 in MDM and induce SAMHD1 dephosphorylation. (A and B) Flow cytometric analysis of MDM infected with replication-competent HIV-1-BAL-HAS (A) and HIV-1ΔEnv-GFP/VSVG (B). The numbers in each graph in the top panel indicate the percentage of events falling within the CD24-positive gate. FSC, forward scatter; APC, allophycocyanin. Data from multiple donors are summarized in the corresponding graph in the bottom panel, with each symbol representing the value for a single donor and mean values from multiple donors provided as a percentage above each condition. Values that are significantly different are indicated by bars and asterisks as follows: **, *P* ≤ 0.01; ***, *P* ≤ 0.001; ****, *P* ≤ 0.0001. Independent one-sample *t* tests were used to determine significance between each IFN treatment condition to the no-IFN control. (C) Lysates (10 µg) from MDM from representative donors (A031 and A049) were analyzed by Western blotting for pSAMHD1-T592, total SAMHD1, and β-actin. Values below each lane indicate the ratio of pSAMHD1 to total SAMHD1 as determined by densitometry. (D) Correlation between pSAMHD1-T592 and percent GFP-positive or (E) total SAMHD1 and percent GFP-positive MDM for donor A031 (circles) and donor A049 (squares). Each symbol represents the mean percentage of infection from triplicate wells as measured by flow cytometry plotted against the ratios determined by Western blotting densitometry, as calculated in Fig. 1C.

We sought to determine whether IFNs from various families could differentially modulate SAMHD1 protein levels or activity and whether these changes would in turn affect HIV-1 infection in macrophages. CD14^+^ monocytes were isolated from peripheral blood mononuclear cells (PBMC) from healthy donors and MDM were generated following 7-day differentiation. MDM were exposed for 24 h to 50-ng/ml concentrations of IFN-α, IFN-ε, IFN-γ, or IFN-λ. Macrophages were infected with a replication-competent R5-tropic recombinant virus (HIV-1-BAL-HSA; see Fig. S1A in the supplemental material) (22) and analyzed for CD24 (mHSA, mouse heat-stable antigen) expression by flow cytometry 48 h later (Fig. 1A). Without IFN treatment, infection levels ranged between 1 and 16% among donors. Because of the high degree of variability in infectivity among donors, we set infection levels in the absence of IFN to 100% for each individual donor and then normalized infection in the presence of IFN to that value (Fig. 1A). In each case, IFN treatment restricted infection, albeit to different degrees: IFN-α, IFN-ε, and IFN-γ potently suppressed infectivity. In contrast, IFN-λ reduced infectivity modestly and in a highly variable manner, a degree of protection that could not be enhanced by increasing concentrations of IFN-λ (Fig. S2A).

10.1128/mBio.00819-18.1 FIG S1  HIV-1 genomes utilized in this study. (A) HIV-1ΔEnv-GFP. (B) HIV-1-BAL-HSA. Download FIG S1, TIF file, 0.1 MB. Copyright © 2018 Szaniawski et al. 2018 Szaniawski et al. This content is distributed under the terms of the Creative Commons Attribution 4.0 International license.

10.1128/mBio.00819-18.2 FIG S2  (A) Dose-response curve of IFN-treated MDM (from donor A008) used to establish working concentrations for experiments in this study. Values indicate percent GFP-positive MDM as measured by flow cytometry 48 h postinfection with HIV-1ΔEnv-GFP/VSVG, which was conducted after 24 h treatment with the indicated IFN. (B) Correlation between CD4 expression and percent GFP-positive macrophages following infection with HIV-1ΔEnv-GFP/VSVG. (C) Correlation between CCR5 expression and percent GFP-positive macrophages following infection with HIV-1ΔEnv-GFP/VSVG. Download FIG S2, TIF file, 5.9 MB. Copyright © 2018 Szaniawski et al. 2018 Szaniawski et al. This content is distributed under the terms of the Creative Commons Attribution 4.0 International license.

To determine whether the effects of IFN are specific to the nature of the envelope glycoprotein, we utilized a replication-defective virus pseudotyped with vesicular stomatitis virus glycoprotein (VSVG) (HIV-1ΔEnv-GFP/VSVG [GFP stands for green fluorescent protein]; Fig. S1B). VSVG binds to the low-density lipoprotein receptor (LDL-R) for entry and shunts the virus toward the endocytic pathway, bypassing membrane fusion resulting from gp160 interaction with chemokine (C-C motif) receptor 5 (CCR5) (23). HIV-1ΔEnv-GFP/VSVG was sensitive to each IFN tested, with IFN-λ exhibiting the least potency and highest donor-to-donor variability. Therefore, we conclude that the observed IFN-stimulated protection was induced against two viruses using distinct envelope glycoproteins (HIV-1 gp160 versus VSVG) that recognize the corresponding receptors (CD4 and CCR5 versus LDL-R) (Fig. 1B).

To examine the potential role of SAMHD1 in restriction by the various IFN types, we generated cell lysates from MDM treated with IFN for 24 h and probed for total SAMHD1 and pSAMHD1-T592 (phosphorylated SAMHD1 [T592 phosphorylated]) (Fig. 1C; two representative donors shown). Addition of recombinant IFN-α, IFN-γ, or IFN-λ led to a reduction in SAMHD1 phosphorylation that correlated directly with reduced infectivity by HIV-1ΔEnv-GFP/VSVG (Fig. 1D). The levels of total SAMHD1 protein were not dramatically affected by any of the IFNs tested, and there was no correlation between total SAMHD1 protein and infectivity after IFN stimulation (Fig. 1E). SAMHD1 activation through T592 dephosphorylation is thus a conserved effector mechanism resulting from stimulation of MDM by different IFN types.

### SAMHD1 is active at baseline and controls MDM resistance to HIV-1.

**FIG 2:**
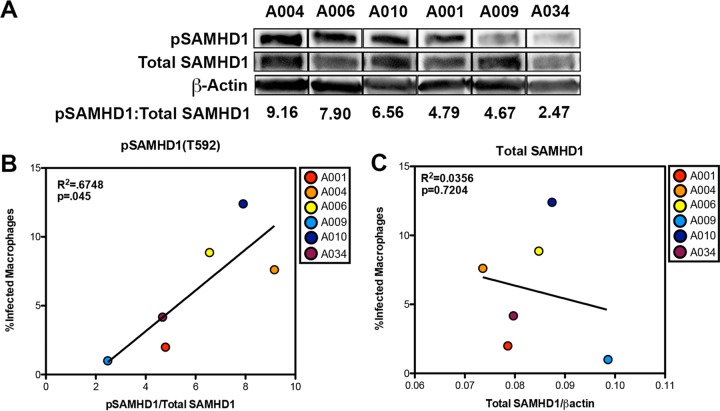
Macrophages exhibit diverse pSAMHD1-T592 signatures at baseline that correlate with infectivity. (A) Lysates of MDM from six donors were analyzed by Western blotting for pSAMHD1-T592, total SAMHD1, and actin. Numbers indicate the proportion of pSAMHD1-T592 to total SAMHD1 as calculated by Western blotting densitometry. Lanes were cut following development of a single membrane to remove alternating lanes. (B) Correlation between pSAMHD1-T592 and the percentage of GFP-positive macrophages as analyzed by flow cytometry. (C) Correlation between total SAMHD1 and the percentage of GFP-positive MDM. Linear regression analyses were performed to study the relationships between infectivity, total SAMHD1 levels, and pSAMHD1 levels.

We hypothesized that highly permissive MDM would exhibit a higher degree of SAMHD1 phosphorylation relative to the levels of total SAMHD1 protein. We derived MDM from six healthy donors and exposed them to HIV-1ΔEnv-GFP/VSVG. In the absence of stimulation, MDM from these donors exhibited vastly different levels of SAMHD1 phosphorylation (Fig. 2A) at baseline, which correlated directly with the levels of infectivity (*P* = 0.045; *R*^2^ = 0.6748; Fig. 2B). Infectivity did not correlate with the levels of total SAMHD1 protein (Fig. 2C). Although a modest correlation between CCR5 cell surface expression and infectivity was observed, differences in infectivity at baseline cannot be attributed to variations in CD4 or CCR5 because the virus used in these experiments was pseudotyped with VSVG (Fig. S2B and C). The finding that MDM from healthy donors exhibit diverse phosphorylation levels of SAMHD1 that correlate directly with infectivity supports our model in which infectivity in MDM depends on SAMHD1 activity and not expression.

### SAMHD1 phosphorylation status is sufficient to explain IFN-induced HIV-1 restriction in primary MDM.

To evaluate whether SAMHD1 is the relevant antiviral effector responsible for the HIV-1 restriction induced by IFNs, we delivered the simian immunodeficiency virus SIVmac accessory protein Vpx to ectopically induce SAMHD1 degradation (6–8, 24–26). To this end, we generated VSVG-pseudotyped virus-like particles (VLP) containing the SIVmac accessory protein Vpx [Vpx(+)VLP] and, as a control, VLP lacking Vpx [Vpx(−)VLP] (8, 24).

**FIG 3:**
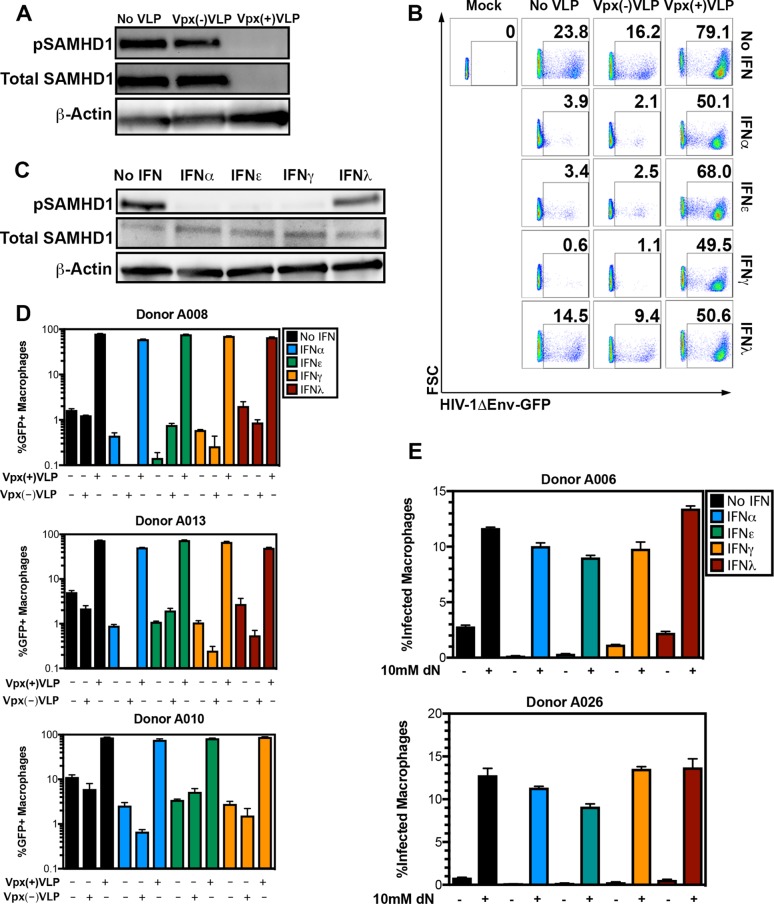
SAMHD1 is the major downstream effector of diverse IFN types. (A) Representative Western blot analysis of MDM extracts not treated with VLP or treated with Vpx(−)VLP and Vpx(+)VLP and probed for pSAMHD1-T592, total SAMHD1, and β-actin. (B) Representative flow cytometric analysis of viable MDM infected with a replication-incompetent HIV-1ΔEnv-GFP/VSVG in the presence or absence of 50 ng/ml IFN-α, IFN-ε, IFN-γ, or IFN-λ (rows) and treated with the Vpx VLP indicated (columns). Numbers in the graphs indicate the percentage of events falling within the GFP-positive gate as established with mock-infected MDM. (C) Western blot analysis of pSAMHD1-T592, total SAMHD1, and actin for macrophages from donor A051. (D) Bar graphs showing macrophages from three additional, independent donors with each condition conducted in triplicate. (E) Macrophages from donors A006 and A026 were treated with IFN, exposed to 10 mM dN (+), and then infected with HIV-1ΔEnv-GFP/VSVG. Bars indicate the percent GFP-positive cells within the viable population as measured by flow cytometry.

Cells were exposed to Vpx(+)VLP or Vpx(−)VLP for 6 h and treated for 18 h with IFN. Cells were then infected with HIV-1ΔEnv-GFP/VSVG. SAMHD1 degradation by Vpx(+)VLP and Vpx(−)VLP was evaluated by Western blotting 24 h after VLP addition, and infection levels were evaluated by flow cytometry 48 h postinfection. Vpx(+)VLP led to efficient SAMHD1 degradation and reversed the block to infection with HIV-1ΔEnv-GFP/VSVG, while Vpx(−)VLP failed to do so (Fig. 3A and B, one representative donor; additional donors shown in Fig. 3D). Importantly, reversal of the IFN-induced restriction in the presence of Vpx(+)VLP was independent of the amount of virus utilized (Fig. S3A). Dephosphorylation of SAMHD1 for one representative donor from this experiment is shown in Fig. 3C. Cells in the absence of IFN also demonstrated increased infectivity when treated with Vpx(+)VLP. These results are consistent with previous reports showing that Vpx can overcome the restriction imposed by SAMHD1 at baseline and in the absence of exogenous IFN stimulation, supporting the notion that SAMHD1 is at least partially active under unstimulated conditions (Fig. 3D) (24).

10.1128/mBio.00819-18.3 FIG S3  (A) Dose-response curves of HIV-1ΔEnv-GFP/VSVG-infected MDM (donors A010 and A062) treated with or without Vpx(+)VLP. (B) Analysis of dCTP in the presence and absence of the indicated IFN. Each symbol represents one unique donor, with each IFN tested in three independent donors. (C) Western blot analysis of pSAMHD1-T592 and CDK1 18 h posttreatment with type I, II, and III IFN. Download FIG S3, TIF file, 23.5 MB. Copyright © 2018 Szaniawski et al. 2018 Szaniawski et al. This content is distributed under the terms of the Creative Commons Attribution 4.0 International license.

To determine whether SAMHD1 activation results in a state of HIV-1 restriction that stems from limiting dNTP levels, we exposed cells to high concentrations of deoxynucleosides (dNs) in the culture medium prior to infection. We predicted that exogenous dN administration would phenocopy the effect of Vpx(+)VLP and reverse the IFN-induced antiviral activity. Deoxyribonucleosides are cell membrane permeable and are converted to dNTPs once inside the cell (6). Addition of dNs to the medium enhanced infection in the absence of IFN and efficiently relieved the IFN-induced restriction in IFN-treated cells (Fig. 3E [two representative donors shown]). These results, together with those in Fig. 3A to D, establish SAMHD1 dephosphorylation as the principal effector mechanism against HIV-1 in the response of MDM to type I, II, and III IFN, which results in dNTP depletion and impaired HIV-1 reverse transcription.

### Canonical HIV-1 restriction factors, but not SAMHD1, are transcriptionally induced by type I, type II, and type III IFN stimulation.

**FIG 4:**
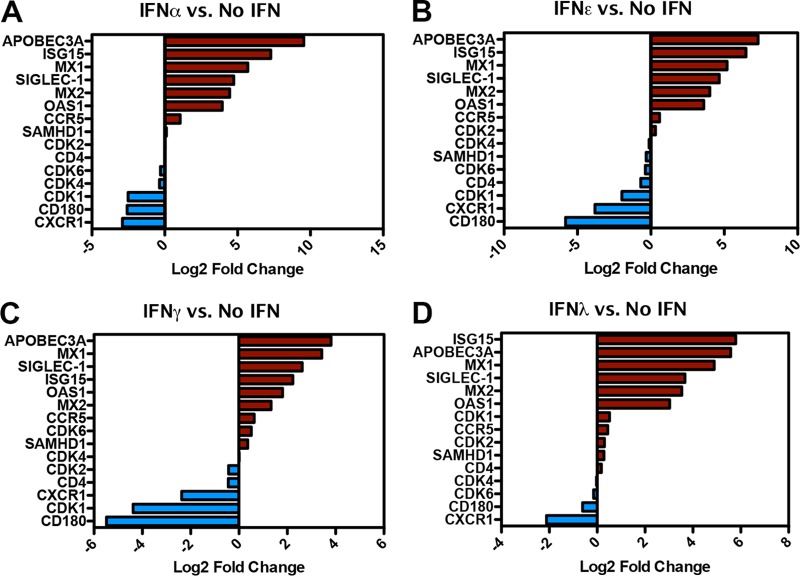
SAMHD1 is not a canonical ISG in MDM. (A to D). Gene expression signatures of select genes represented as log_2_ fold change relative to the untreated condition (no IFN) for IFN-α, IFN-ε, IFN-γ, and IFN-λ.

To further explore and compare the cellular responses to type I, II, and III IFN, we performed high-throughput RNA sequencing (RNA-seq) with MDM stimulated with IFN-α, IFN-ε, IFN-γ, or IFN-λ. mRNA was isolated 18 h after stimulation and subjected to RNA-seq. Figure 4 shows the log_2_ fold change (sequence reads in IFN treatment over those without IFN) in mRNA for SAMHD1 along with a small subset of known interferon-stimulated genes (ISGs) (ISG15, ABOBEC3A, SIGLEC-1, MX1, MX2, and OAS1), cell cycle regulators known to act on SAMHD1 (cyclin-dependent kinase 1 [CDK1], -2, -4, and -6) and immune-related genes (CD4, CCR5, chemokine [C-X-C motif] receptor 1 [CXCR1], and CD180). SAMHD1 mRNA levels were not dramatically affected by any of the IFN treatments (1.08-fold [IFN-α], −1.24-fold [IFN-ε], 1.27-fold [IFN-γ], and 1.21-fold [IFN-λ]), in agreement with previous reports in CD4^+^ T cells and dendritic cells (27). In contrast, many ISGs were strongly induced by each of the IFNs, exhibiting expression levels that ranged between 5-fold (ISG15 by IFN-γ) and 750-fold (APOBEC3A by IFN-α) over the no-IFN condition (Fig. 4A to D).

**FIG 5:**
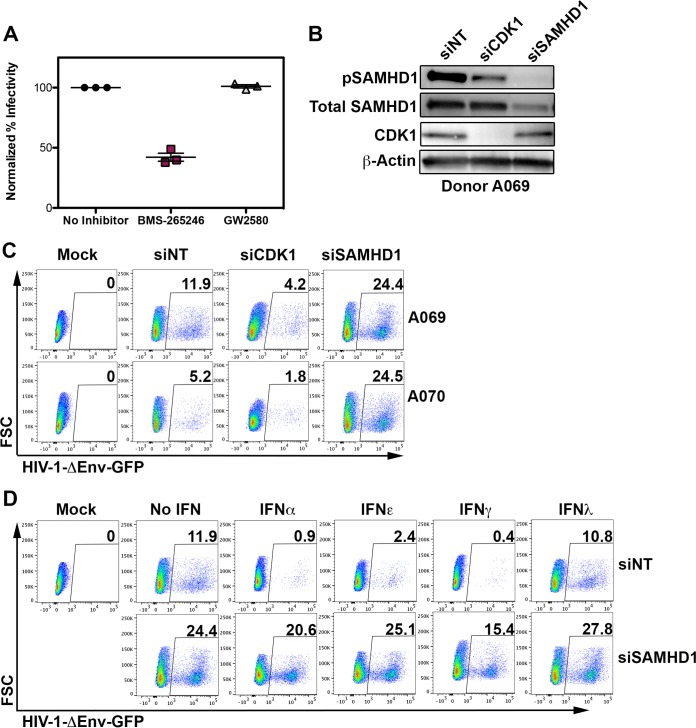
siRNA to CDK1 and SAMHD1 reveal central roles in IFN-induced HIV-1 restriction. (A) The effect of CDK1/2 inhibition on infection with HIV-1ΔEnv-GFP/VSVG was determined by flow cytometry, with infection in untreated cells set at 100%. Data on cells from three representative donors are shown. (B) Western blot analysis of siRNA-treated MDM from donor A069. (C) Representative siRNA flow cytometry plots from two donors, donors A069 and A070. (D) Representative flow cytometry plots from IFN-treated MDM with and without SAMHD1 knockdown.

Among the cyclin-dependent kinases examined in our RNA-seq experiment, CDK1 mRNA was decreased 5.7-fold, 3.9-fold, and 20.8-fold after IFN-α, IFN-ε, and IFN-γ, respectively (Fig. 4A to C). CDK1 transcripts were modestly upregulated by IFN-λ (1.4-fold), the only IFN with modest to undetected HIV-1 restriction in our studies. Therefore, we speculated that downregulation of CDK1 by IFN-α, IFN-ε, and IFN-γ is part of the mechanism by which these IFNs induce dephosphorylation of SAMHD1. To verify this prediction, we tested the CDK1/CDK2 inhibitor BMS-265246 for its ability to restrict HIV-1ΔEnv-GFP/VSVG infection. BMS-265246 restricted HIV-1 infection in MDM, whereas GW2580, a tyrosine kinase inhibitor (TKI) that targets colony-stimulating factor 1 receptor (CSF-1R) among other receptor tyrosine kinases (RTK), had no effect on viral infectivity (Fig. 5A) (28).

We next used a small interfering RNA (siRNA) to CDK1 to verify its role in SAMHD1 phosphorylation and HIV-1 restriction. Knockdown of CDK1 resulted in a significant reduction in SAMHD1 phosphorylation, concomitant with a reduction in MDM susceptibility to HIV-1, while the use of a siRNA targeting SAMHD1 led to an enhanced susceptibility of MDM to HIV-1 (Fig. 5B and C). In agreement with our findings with Vpx(+)VLP, siRNA-mediated knockdown of SAMHD1 similarly reversed the IFN-induced restriction to HIV-1, suggesting that the majority of the antiviral effect is exerted through SAMHD1 activation (Fig. 5D).

### SAMHD1 activity can be regulated pharmacologically.

**FIG 6:**
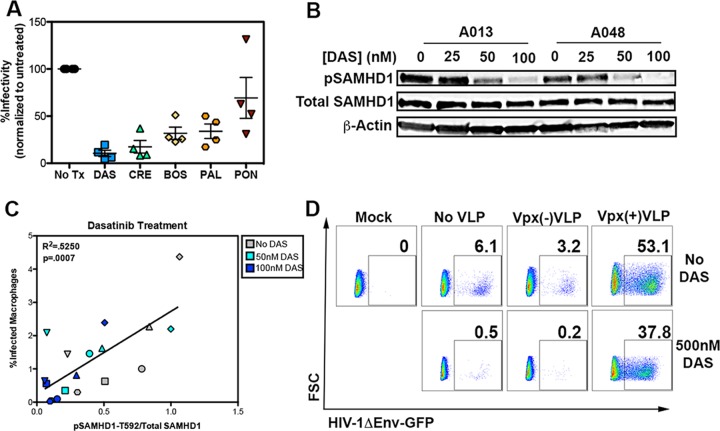
SAMHD1 is required for TKI-induced restriction. (A) Summary flow cytometric analysis of HIV-1 infection in four separate donors treated with various TKIs. The TKIs were dasatinib (DAS), crenolanib (CRE), bosutinib (BOS), palbociclib (PAL), and ponatinib (PON). Values indicate the percent GFP-positive cells within the viable population of cells infected with HIV-1ΔEnv-GFP/VSVG. The viability of TKI-treated cells is between 90 and 100% that of untreated cells (No Tx). (B) Western blot analysis of lysates of MDM from two representative donors treated with the indicated concentration of dasatinib. (C) Correlation between relative pSAMHD1-T592 and infectivity for macrophages from six donors treated with 0 nM, 50 nM, and 100 nM dasatinib. (D) MDM treated with 500 nM dasatinib and treated with the Vpx VLP indicated. Numbers represent the percentage of live cells within the GFP-positive gate as established with mock-infected MDM. Linear regression analysis was performed to study the relationship between infectivity and pSAMHD1 levels during dasatinib treatment.

Because activation of SAMHD1 imposes a potent blockade against HIV-1, it would be ideal if the activity of SAMHD1 could be controlled pharmacologically. Recently, it was shown that this can be accomplished in CD4^+^ T cells with the use of dasatinib, a Bcr-Abl-specific TKI that has been approved for use in chronic myelogenous leukemia (CML) and Philadelphia chromosome-positive acute lymphoblastic leukemia (Ph+ AML) by the FDA (29, 30). We tested a panel of tyrosine kinase inhibitors to determine whether they possess anti-HIV-1 activity and whether that activity stems from SAMHD1 dephosphorylation. Among the TKIs tested were dasatinib, bosutinib, crenolanib, palbociclib, and ponatinib. These compounds exhibit low cytotoxicity, are FDA approved, and target a variety of cellular pathways, including Abl kinase, Src family kinases, type III RTK, as well as CDK4 and CDK6 (31). Each inhibitor led to HIV-1 restriction, with dasatinib providing the most potent HIV-1 blockade (Fig. 6A). HIV-1ΔEnv-GFP/VSVG infectivity was inversely correlated with the proportion of SAMHD1 present in its activated, dephosphorylated state as manipulated by the TKIs (Fig. 6B and C). We focused on dasatinib to test whether SAMHD1 phosphorylation and infectivity exhibited a dose-dependent relationship and observed overt changes in pSAMHD1-T592 that corresponded to increasing concentrations of the inhibitor (Fig. 6B and C). The dephosphorylation of SAMHD1 was concomitant with and inversely proportional to the infectivity by HIV-1ΔEnv-GFP/VSVG (Fig. 6C).

TKIs, including dasatinib, target diverse cellular pathways at high nanomolar concentrations (32). If SAMHD1 is indeed responsible for the effects of the TKIs tested, then incubation with Vpx(+)VLP following treatment should induce degradation of SAMHD1 and overcome the TKI-imposed restriction. Incubation with dasatinib rendered cells highly resistant to infection (0.5%) compared with untreated cells (6.0%), representing a 92% protection (Fig. 6D). Addition of Vpx(+)VLP to untreated cells enhanced infection 8.8-fold (6.0% to 53.0%), while addition of Vpx(+)VLP in the context of dasatinib enhanced infection 76-fold (0.5% to 38%), representing a reversal of the dasatinib-induced antiviral state. Taken together, our experiments establish a causal link between SAMHD1 activation and protection from HIV-1 infectivity in MDM and provide evidence supporting a dynamic regulatory role for SAMHD1 in MDM. Furthermore, we demonstrate that SAMHD1 activity can be manipulated pharmacologically to render macrophages refractory to HIV-1 infection.

## DISCUSSION

Our experiments demonstrate that SAMHD1 dephosphorylation at threonine-592 represents a central mechanism of HIV-1 restriction that is common to the three known families of IFNs, though with vastly different efficiencies, as evidenced by the limited potency of IFN-λ. Enhanced dNTPase activity appears to be the functional outcome of SAMHD1 activation, supported by several lines of evidence, including complete reversal of the IFN-induced restriction by the addition of exogenous dNs and degradation of SAMHD1 by the SIV protein Vpx (8, 24). The finding that diverse IFNs are functional to the degree to which they modulate SAMHD1 phosphorylation and activation without effecting changes in total protein is an important step in understanding innate immune responses in macrophages. We also clarify that the previously reported antiviral activity of IFN-ε is associated with dephosphorylation of SAMHD1 similar to what has been described for other type I IFNs (17). Finally, we show that HIV-1 inhibition by various FDA-approved TKIs is dependent on the activation of SAMHD1, providing important mechanistic understanding as to how these compounds act and can be directed toward HIV-1 cure efforts.

Macrophages are important targets of HIV-1 infection *in vivo* and are key players in infection establishment and viral persistence (33, 34). It has been speculated that macrophages can support HIV-1 infection and harbor virus over prolonged periods of time independent of T cells, even in the setting of ART, a hypothesis that has recently been strengthened by experiments conducted in humanized myeloid-only mice (1, 35). Therefore, strategies that aim to prevent virus spread to tissue macrophages, either independently or in the context of latency reversal, will be important components of ongoing HIV-1 cure efforts.

SAMHD1 was first identified as the human homolog of a previously described mouse IFN-γ-inducible GTP-binding protein known as MG11 (36, 37). Recently, SAMHD1 has been shown to be induced by stimulation with type I and type II IFNs via downregulation of miR-181 and miR-30a in human monocytes (38). A similar phenomenon was observed in astrocytes and microglia and was also dependent on miR181a (39). In hepatocytes, it has been shown that type I and type II IFN can induce SAMHD1 transcription, inducing an antiviral state that restricts hepatitis B virus (HBV) infection (40). Additionally, it has been shown that in mature dendritic cells (DCs), coculture with lymphocytes can lead to downregulation of SAMHD1 and enhance DC permissiveness to HIV-1 (41). In the present study, we show that the type I, II, and III IFN-induced HIV-1 restriction in MDM is not derived from changes in SAMHD1 protein or mRNA levels and that the antiviral state hinges upon changes in SAMHD1 activity as determined by T592 phosphorylation.

The activity of SAMHD1 in lymphocytes is regulated by cyclin/cyclin-dependent kinase (CDK)-mediated threonine-592 phosphorylation in a cell cycle-dependent manner (21). However, SAMHD1 phosphorylation can also be regulated independent of cell division (42). The specific CDKs responsible for SAMHD1 kinase activity depend on cell type, with CDK1, CDK2, CDK4, and CDK6 all demonstrating a regulatory effect on SAMHD1 in different contexts (21, 42–44). Phosphorylation of residue T592 impairs SAMHD1 tetramerization resulting in diminished capacity for dNTP hydrolysis and impaired anti-HIV-1 activity (45, 46). Our results suggest that IFN-induced activation of SAMHD1 is effected via downregulation of CDK1 mRNA and indicate that in the absence of stimulation, CDK1 maintains SAMHD1 phosphorylated and, to a large extent, inactive. However, CDK1 cannot be the sole regulator of SAMHD1 in MDM, as we also observed a modest downregulation of CDK1 protein by IFN-λ, which was not accompanied by significant protection from viral infection. Therefore, additional factors, such as phosphorylation of CDK1, or the action of an IFN-regulated phosphatase, need to be identified.

The finding that diverse IFNs converge on SAMHD1 activation reveals a common denominator in IFN signaling, despite differences in expression of numerous other documented antiviral genes in response to IFN stimulation (10–13). Whether restriction factors other than SAMHD1 were at play in our studies is unclear, though plausible. In fact, a recent study showed that SIV Vpx of red-capped mangabeys and mandrills (SIVrcm/mnd-2) enhances HIV-1 infection specifically in resting CD4^+^ T cells in a SAMHD1- and dNTP pool-independent manner and led the authors to suggest the existence of an unknown restriction factor (47).

Importantly, the present study utilized two major virus clones, both of which are derived from the lab-adapted, NL4-3 infectious clone (48). It is known that viruses present during the acute phase of infection, termed transmitted-founder (T/F) viruses, exhibit enhanced resistance to type I IFN than those present during chronic infection (chronic carrier [C/C]) (49, 50). HIV-1-BAL-HSA, a hybrid virus composed of HIV-1-BAL envelope expressed on a lab-adapted NL4-3 background, showed a trend in IFN susceptibility similar to those observed with our NL4-3-derived HIV-1ΔEnv-GFP/VSVG construct (22). Future studies aimed at delineating the requirement of SAMHD1 activation for diverse IFN function in the setting of T/F and C/C HIV-1 strains will be important in further understanding the biology of infection of macrophages with HIV-1, specifically with respect to those IFNs expressed in mucosal sites of transmission (51, 52).

SAMHD1 has recently been proposed as a potential tumor suppressor, and in fact, many FDA-approved anti-cancer TKIs have potent effects on SAMHD1 activation *in vitro* and *in vivo* (29, 30, 44). In cancer, SAMHD1 is thought to play a role in controlling cell cycle of tumor cells, where its activity as a triphosphohydrolase can restrain uncontrolled cellular proliferation by blunting cellular DNA synthesis (53, 54). Expanding our study beyond the natural biology of HIV-1 infection, we show that SAMHD1 activity can be targeted by several FDA-approved anti-cancer TKIs. TKIs exerted their anti-HIV-1 activity through threonine (T592) dephosphorylation, which is unusual. We speculate that TKIs inhibit a signaling cascade initiated by TK culminating in the regulation of Ser/Thr kinases, including CDK1. Future studies aimed at delineating these pathways will be important in understanding how SAMHD1 activity is controlled at steady state and can be manipulated therapeutically. Targeting the relevant pathways may prove useful in anti-HIV-1 efforts or in preventing end organ damage observed in patients on ART, including HIV-1-associated neurologic dysfunction.

## MATERIALS AND METHODS

### Isolation of healthy donor PBMC.

Healthy donors 18 years old and older were recruited for this study under the University of Utah Institutional Review Board (IRB) protocol 67637. Written informed consent was obtained from all donors. Whole blood was obtained by peripheral phlebotomy, and peripheral blood mononuclear cells (PBMC) were isolated using a Lymphoprep density gradient (Stemcell Technologies).

### Generation and infection of MDM.

CD14^+^ monocytes were isolated via positive selection with magnetic beads (Miltenyi Biotec). Cells were allowed to adhere in serum-free medium for 2 h, which was then removed and replaced with RPMI 1640 medium supplemented with 10% pooled human serum (Innovative Research). The medium was changed at day 5, and cells were cultured for a total of 7 days to allow differentiation to MDM prior to experimentation as previously described. MDM were infected with 250 ng of either HIV-1-BAL-HSA or HIV-1ΔEnv-GFP/VSVG as determined by p24 enzyme-linked immunosorbent assay (ELISA) for 6 h. Cells were washed twice with fresh medium to remove unbound virus. Infection was quantified via flow cytometry at 48 h postinfection.

### Generation of viruses.

Replication-defective virus (HIV-1ΔEnv-GFP/VSVG) was generated using calcium phosphate-mediated transfection of HEK293T cells. Briefly, HIV-1ΔEnv-GFP and VSVG plasmids were cotransfected for 6 h. The transfection medium was removed, and cells were cultured over 2 days, with virus-containing supernatants removed at 24 and 48 h posttransfection. These viruses contain a frameshift mutation in envelope and are capable of only a single-round infection when Env is provided in *trans* as previously described (55). Replication-competent virus (HIV-1-BAL-HSA) was generated through a similar transfection protocol using a single plasmid (pNL-43-BAL-IRES-HSA) courtesy of Michel Tremblay (Centre Hospitalier de l’Université Laval). All viruses were quantified using p24 ELISA (Zeptometrix) and stored at −80 °C until further use.

### RNA-seq analysis for ISGs.

MDM were generated as described and stimulated with 25 ng/ml of the indicated IFNs. Total RNA was isolated 18 h following stimulation using RNeasy minikit (Qiagen). Intact poly(A) RNA was purified from total RNA samples (100 to 500 ng) with oligo(dT) magnetic beads, and stranded mRNA sequencing libraries were prepared as described using the Illumina TruSeq Stranded mRNA Library Preparation kit (catalog no. RS-122-2101 and RS-122-2102). Purified libraries were qualified on an Agilent Technologies 2200 TapeStation using a D1000 ScreenTape assay (catalog no. 5067-5582 and 5067-5583). The molarity of adapter-modified molecules was defined by quantitative PCR using the Kapa Biosystems Kapa Library Quant kit (catalog no. KK4824). Individual libraries were normalized to 10 nM, and equal volumes were pooled in preparation for Illumina sequence analysis. Sequencing libraries (25 pM) were chemically denatured and applied to an Illumina HiSeq v4 single-read flow cell using an Illumina cBot system. Hybridized molecules were clonally amplified and annealed to sequencing primers with reagents from an Illumina HiSeq SR cluster kit v4-cBot (catalog no. GD-401-4001). Following transfer of the flow cell to an Illumina HiSeq 2500 instrument (HCSv2.2.38 and RTA v1.18.61), a 50-cycle single-read sequence run was performed using HiSeq SBS kit v4 sequencing reagents (catalog no. FC-401-4002).

### Inhibitors and IFNs.

Recombinant human IFN-α (PBL Assay Science), IFN-ε (EnQuire Bio), IFN-γ (PeproTech), and IFN-λ (PeproTech) were purchased from suppliers, resuspended at 50 ng/µl, and added to cell culture at 50 ng/ml, the optimal concentration for HIV-1 restriction determined empirically in our cell culture system. The following units of each IFN tested were used: 7,850 U/test for IFN-α, 500 U/test for IFN-γ, and 125 U/test for IFN-λ. IFN-ε specific activity was untested by the manufacturer and thus utilized at a mass concentration identical to IFN-α, IFN-γ, and IFN-λ. Inhibitors were purchased from suppliers and resuspended at a concentration of 1 mM and added to culture at a concentration of 1 µM.

### siRNA knockdown of CDK1 and SAMHD1.

MDM were transfected twice (24 h apart) with either control siRNA or siRNAs against SAMHD1 or CDK1 at a final concentration of 111 nM using Lipofectamine RNAiMAX (Thermo Fisher) according to the manufacturer’s protocol. At 6 h posttransfection, cells were supplemented with 1 volume of RPMI 1640 medium supplemented with 10% pooled human serum. Twenty-four hours after the second transfection, the cells were either mock infected or infected with HIV-1ΔEnv-GFP/VSVG. Forty-eight hours later, MDM were analyzed for green fluorescent protein (GFP) expression via flow cytometry or lysed and analyzed via Western blotting and probed for SAMHD1, pSAMHD1, and CDK1 expression.

### Quantification of cellular deoxynucleoside triphosphates (dNTPs).

Samples were extracted by adding 90 µl of an ice-cold 60% solution of methanol (aqueous) which contained 0.1 µg/ml of ^13^C10-^15^N5 ATP (Sigma-Aldrich) as an internal standard. Each sample was vortexed for 10 s and chilled at −20°C prior to being centrifuged for 10 min at 20,000 relative centrifugal force (RCF) at 4°C. The supernatant was removed to a fresh tube and dried overnight *en vacuo*. Immediately prior to liquid chromatography-mass spectrometry (LC-MS) analysis, the samples were reconstituted in 50 µl water containing 10 mM ammonium carbonate.

Mass spectral analysis was performed using an Agilent 6490 ultraperformance liquid chromatography (UPLC) triple-quadrupole (QQQ) MS system (Santa Clara, CA). A SeQuant ZIC hydrophilic interaction liquid chromatographic (HILIC) column (Millipore Sigma, Burlington, MA) was used for fractionation using a linear gradient of 10:90 acetonitrile (ACN)–10 mM ammonium acetate buffer to 20:80 ACN–10 mM ammonium acetate buffer over 20 min at a flow rate of 0.2 ml/min. Mass spectrometry was performed in the positive mode with an Agilent jet stream (AJS) electrospray ionization (ESI) source using tandem mass spectrometry (MS/MS) fragmentation. The deoxynucleotides were positively identified by both chromatographic retention time, and the ion ratios were identified by at least two selected reaction monitoring (SRM) transitions. Quantitative data analysis was conducted using Agilent MassHunter Quant software for dCTP using the internal standard to normalize signal between samples.

### Statistical analysis.

All statistical analyses were performed using GraphPad Prism 5.0 (GraphPad Software, San Diego, CA).
