# Supplementary figures and images for: SAMHD1 Phosphorylation Coordinates the Anti-HIV-1 Response by Diverse Interferons and Tyrosine Kinase Inhibition
Source: mBio. 2018 May 15;9(3):e00819-18. doi: 10.1128/mBio.00819-18 (PMC5954222; doi:10.1128/mBio.00819-18)

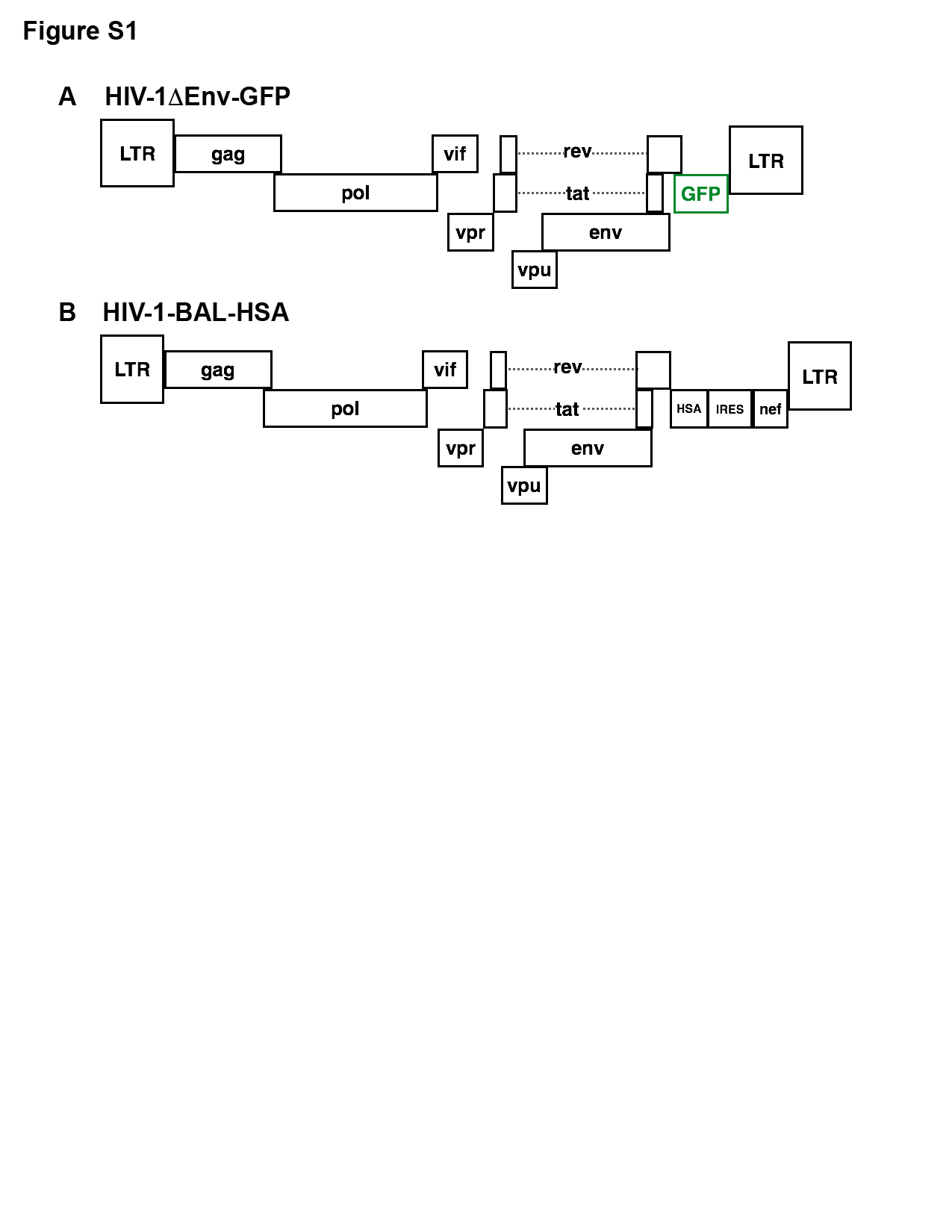

Supplement: FIG S1 [file mbo003183891sf1.tif]

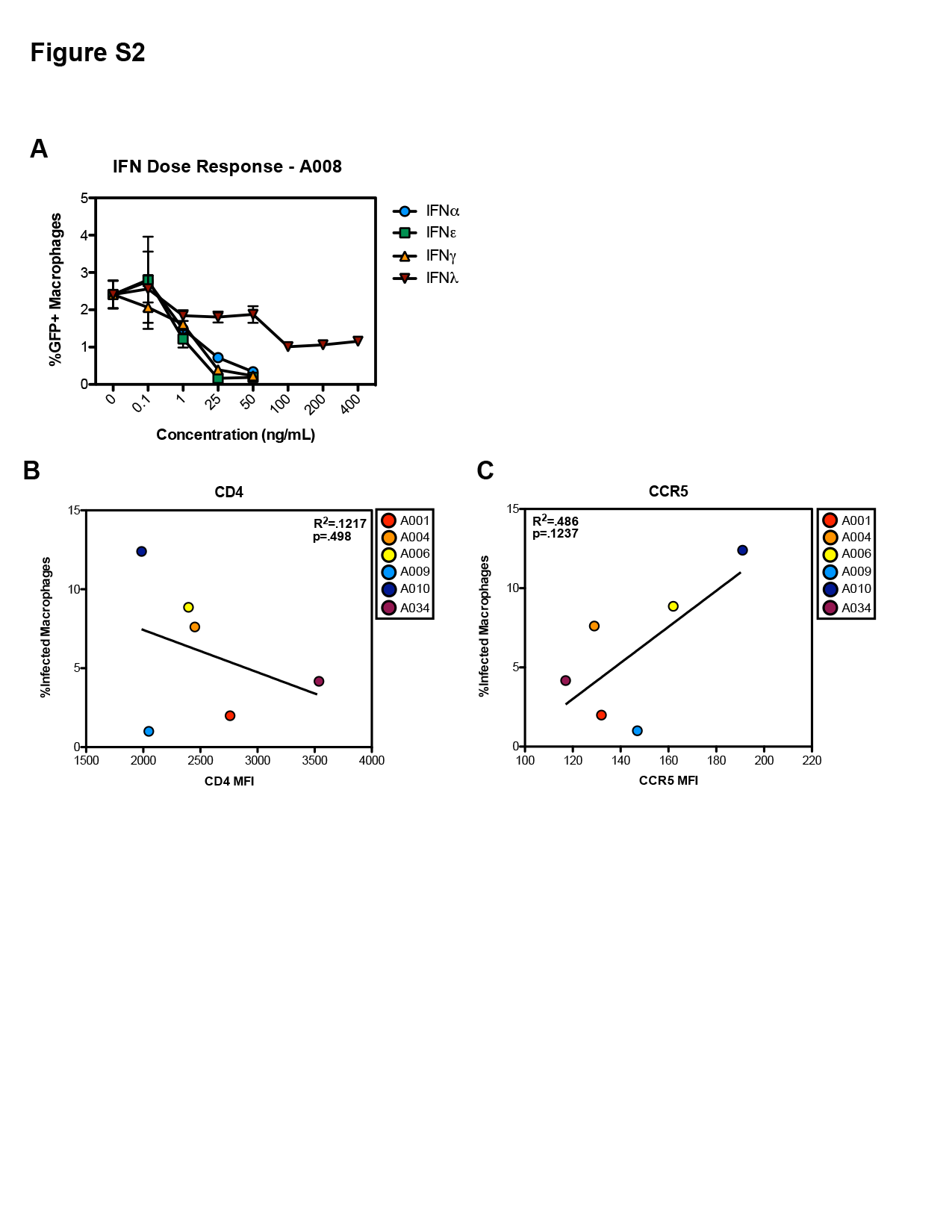

Supplement: FIG S2 [file mbo003183891sf2.tif]

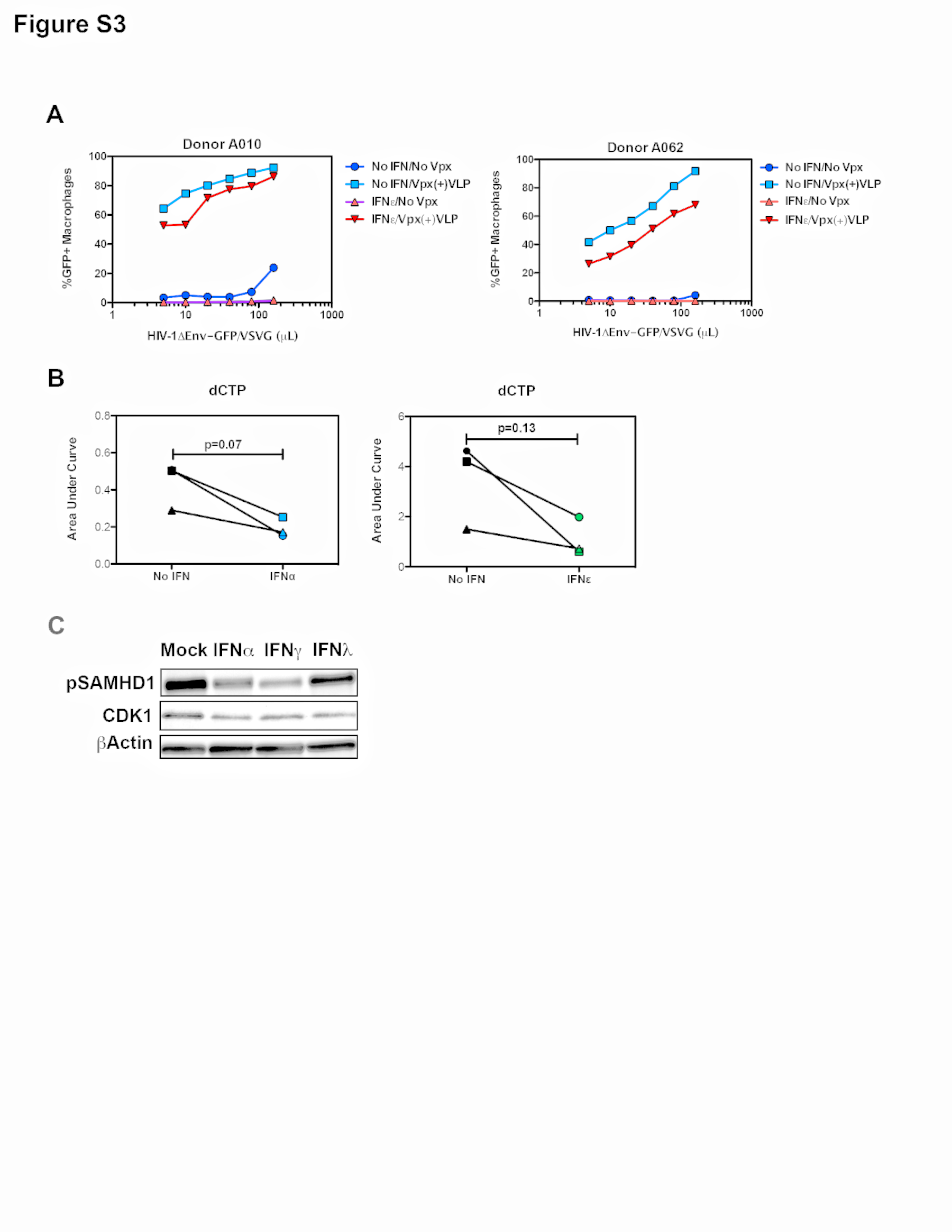

Supplement: FIG S3 [file mbo003183891sf3.tif]
